# 3-[(3*S*)-3-Ethyl-1-methyl­azepan-3-yl]phenyl *N*-(4-fluoro­phen­yl)carbamate

**DOI:** 10.1107/S1600536810024396

**Published:** 2010-06-26

**Authors:** Jian Sun, Qiong Xie, Zhui-Bai Qiu

**Affiliations:** a Department of Medicinal Chemistry, School of Pharmacy, Fudan University, 826 Zhangheng Road, Shanghai 201203, People’s Republic of China

## Abstract

The asymmetric unit of the title compound, C_22_H_27_FN_2_O_2_, a (−)-*S*-meptazinol derivative, contains two mol­ecules. The azepane ring adopts a similar twist chair form in both mol­ecules, while the dihedral angles between the two benzene rings are 88.17 (14) and 89.93 (14)° in the two mol­ecules. The absolute configuration of the mol­ecule was determined from the synthetic starting material. The crystal structure is stabilized by classical inter­molecular N—H⋯O hydrogen bonds.

## Related literature

For a related structure, see: Ennis *et al.* (1986 ▶).
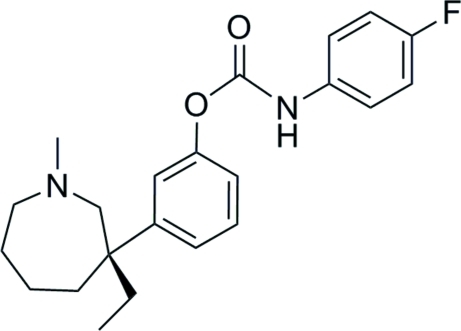


## Experimental

### 

#### Crystal data

C_22_H_27_FN_2_O_2_
*M* *_r_* = 370.46
Monoclinic, 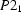

*a* = 11.3836 (17) Å
*b* = 9.7368 (15) Å
*c* = 19.008 (3) Å
β = 105.564 (3)°
*V* = 2029.6 (5) Å^3^
*Z* = 4
Mo *K*α radiation
μ = 0.08 mm^−1^
*T* = 295 K
0.30 × 0.28 × 0.21 mm

#### Data collection

Bruker SMART CCD area-detector diffractometer
Absorption correction: multi-scan (*SADABS*; Sheldrick, 1996 ▶) *T*_min_ = 0.565, *T*_max_ = 1.000
10768 measured reflections
7172 independent reflections
3625 reflections with *I* > 2σ(*I*)
*R*_int_ = 0.092

#### Refinement

*R*[*F* ^2^ > 2σ(*F* ^2^)] = 0.058
*wR*(*F* ^2^) = 0.134
*S* = 0.83
7172 reflections
437 parameters
1 restraint
H-atom parameters constrained
Δρ_max_ = 0.26 e Å^−3^
Δρ_min_ = −0.20 e Å^−3^

### 

Data collection: *SMART* (Bruker, 2001 ▶); cell refinement: *SAINT* (Bruker, 2001 ▶); data reduction: *SAINT*; program(s) used to solve structure: *SHELXS97* (Sheldrick, 2008 ▶); program(s) used to refine structure: *SHELXL97* (Sheldrick, 2008 ▶); molecular graphics: *SHELXTL* (Sheldrick, 2008 ▶); software used to prepare material for publication: *SHELXTL*.

## Supplementary Material

Crystal structure: contains datablocks I, global. DOI: 10.1107/S1600536810024396/rk2204sup1.cif

Structure factors: contains datablocks I. DOI: 10.1107/S1600536810024396/rk2204Isup2.hkl

Additional supplementary materials:  crystallographic information; 3D view; checkCIF report

**Fig. 1.**
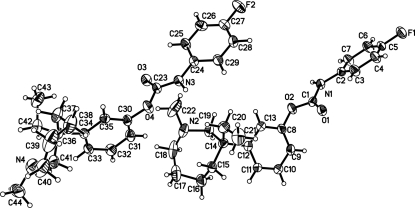
The molecular structure of title compound with the atom numbering scheme. The displacement ellipsoids are drawn at the 50% probability level. H atoms are presented as a small spheres of arbitrary radius.

Enhanced figure: interactive version of Fig. 1

## Figures and Tables

**Table 1:** Hydrogen-bond geometry (Å, °)

*D*—H⋯*A*	*D*—H	H⋯*A*	*D*⋯*A*	*D*—H⋯*A*
N3—H3*A*⋯O3^i^	0.86	2.15	3.007 (5)	177
N1—H1⋯O1^ii^	0.86	2.16	3.002 (5)	167

Symmetry codes: (i) 
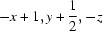
; (ii) 
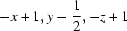
.

## supplementary crystallographic information

### Comment

(-)–Meptazinol is a moderate *AChE* inhibitor (Ennis *et al.*, 1986). Its absolute configuration was determined as *S* by X–ray crystal structures. The title compound is derived from (-)–*S*–meptazinol. We report here the molecular structure of the title compound in order to study the relationship between its structure and *AChE* inhibitory activity.

The molecular structure of the title compound presented on Fig. 1. The asymmetric unit has two molecules. The azepane ring adopts a similar twist chair form in both molecules. The dihedral angle between of two benzene rings is 88.17 (14)° and 89.93 (14)° in the first and second molecules respectively. The crystal structure is stabilized by classical intermolecular hydrogen bonds - N3—H3A···O3^i^ and N1—H1···O1^ii^. Symmetry codes: (i) -*x*+1, *y*+1/2, -*z*; (ii) -*x*+1, *y*-1/2, -*z*+1.

The absolute configuration of the azepane ring atom is C14(*S*), since the title compound was synthesized from (-)–*S*–meptazinol by esterification of the phenol group, which does not interfere with the chiral center C14.

### Experimental

(-)–Meptazinol (200 mg, 0.86 mmol) was dissolved in anhydrous ether (5 ml) and a piece of Na metal (approximately 10 mg) was added. The mixture was stirred at room temperature for 30 min. Then 4–fluorophenylisocyanate (230 mg, 1.72 mmol) was added. The reaction mixture was continuously stirred for 4 h at room temperature and monitored by *TLC*. The precipitate was filtered off and the filtrate was evaporated to give yellow oil. The 10 ml H_2_O was added and pH of the aqueous layer was adjusted to 3 by 1*N* HCl, washed with *Et*_2_O, and then pH was adjusted to 10 by saturated Na_2_CO_3_ aqueous solution. The resulting precipitate was filtered and washed with water three times. A white solid (yield 200 mg, 65.5%) was obtained. Single crystals suitable for X–ray analysis were obtained by slow evaporation of an acetonitrile solution.

### Refinement

All H atoms were placed in the idealized positions with C—H = 0.93–0.96Å, and N—H = 0.86Å. The *U*_iso_(H) = 1.2–1.5 *U*_eq_(C,N).

The 3164 Friedel pairs were merged in crystal refinement procedure.

### Crystal data

**Table:**

C_22_H_27_FN_2_O_2_	*F*(000) = 792
*M**_r_* = 370.46	*D*_x_ = 1.212 Mg m^−^^3^
Monoclinic, *P*2_1_	Mo *K*α radiation, λ = 0.71073 Å
Hall symbol: P 2yb	Cell parameters from 1695 reflections
*a* = 11.3836 (17) Å	θ = 4.5–41.4°
*b* = 9.7368 (15) Å	µ = 0.08 mm^−^^1^
*c* = 19.008 (3) Å	*T* = 295 K
β = 105.564 (3)°	Prism, colourless
*V* = 2029.6 (5) Å^3^	0.30 × 0.28 × 0.21 mm
*Z* = 4	

### Data collection

**Table:**

Bruker SMART CCD area-detector diffractometer	7172 independent reflections
Radiation source: fine–focus sealed tube	3625 reflections with *I* > 2σ(*I*)
graphite	*R*_int_ = 0.092
φ and ω scans	θ_max_ = 25.5°, θ_min_ = 1.9°
Absorption correction: multi-scan (*SADABS*; Sheldrick, 1996)	*h* = −13→10
*T*_min_ = 0.565, *T*_max_ = 1.000	*k* = −11→11
10768 measured reflections	*l* = −23→22

### Refinement

**Table:**

Refinement on *F*^2^	Primary atom site location: structure-invariant direct methods
Least-squares matrix: full	Secondary atom site location: difference Fourier map
*R*[*F*^2^ > 2σ(*F*^2^)] = 0.058	Hydrogen site location: inferred from neighbouring sites
*wR*(*F*^2^) = 0.134	H-atom parameters constrained
*S* = 0.83	*w* = 1/[σ^2^(*F*_o_^2^) + (0.0436*P*)^2^] where *P* = (*F*_o_^2^ + 2*F*_c_^2^)/3
7172 reflections	(Δ/σ)_max_ = 0.041
437 parameters	Δρ_max_ = 0.26 e Å^−^^3^
1 restraint	Δρ_min_ = −0.20 e Å^−^^3^

### Special details

**Table:**

Geometry. All s.u.'s (except the s.u. in the dihedral angle between two l.s. planes) are estimated using the full covariance matrix. The cell s.u.'s are taken into account individually in the estimation of s.u.'s in distances, angles and torsion angles; correlations between s.u.'s in cell parameters are only used when they are defined by crystal symmetry. An approximate (isotropic) treatment of cell s.u.'s is used for estimating s.u.'s involving l.s. planes.
Refinement. Refinement of *F*^2^ against ALL reflections. The weighted *R*–factor *wR* and goodness of fit *S* are based on *F*^2^, conventional *R*–factors *R* are based on *F*, with *F* set to zero for negative *F*^2^. The threshold expression of *F*^2^ > σ(*F*^2^) is used only for calculating *R*–factors(gt) *etc*. and is not relevant to the choice of reflections for refinement. *R*–factors based on *F*^2^ are statistically about twice as large as those based on *F*, and *R*–factors based on ALL data will be even larger.

### Fractional atomic coordinates and isotropic or equivalent isotropic displacement parameters (Å^2^)

**Table:**

	*x*	*y*	*z*	*U*_iso_*/*U*_eq_	
F1	0.0700 (2)	0.6112 (3)	0.22631 (14)	0.0913 (10)	
F2	0.0725 (2)	0.3412 (3)	−0.27982 (14)	0.0860 (10)	
N1	0.4300 (3)	0.4547 (4)	0.46958 (18)	0.0523 (10)	
H1	0.4384	0.3671	0.4741	0.063*	
N2	0.7713 (4)	0.3080 (5)	0.9169 (2)	0.0838 (12)	
N3	0.4376 (3)	0.4940 (4)	−0.03739 (18)	0.0496 (10)	
H3A	0.4511	0.5810	−0.0344	0.060*	
N4	0.8545 (5)	0.6054 (5)	0.4366 (2)	0.0949 (15)	
O1	0.5086 (3)	0.6544 (3)	0.52648 (15)	0.0577 (8)	
O2	0.5776 (3)	0.4446 (3)	0.57009 (16)	0.0688 (10)	
O3	0.5055 (3)	0.2960 (3)	0.02499 (16)	0.0618 (9)	
O4	0.5764 (3)	0.5055 (3)	0.06587 (15)	0.0621 (9)	
C1	0.5050 (4)	0.5303 (5)	0.5213 (2)	0.0454 (12)	
C2	0.3388 (2)	0.5017 (3)	0.40860 (12)	0.0481 (11)	
C3	0.2979 (2)	0.4082 (2)	0.35204 (15)	0.0642 (14)	
H3	0.3315	0.3206	0.3555	0.077*	
C4	0.2069 (3)	0.4458 (3)	0.29030 (13)	0.0732 (15)	
H4	0.1796	0.3832	0.2525	0.088*	
C5	0.1568 (2)	0.5767 (3)	0.28511 (13)	0.0612 (13)	
C6	0.1976 (3)	0.6702 (3)	0.34168 (17)	0.0723 (15)	
H6	0.1641	0.7578	0.3382	0.087*	
C7	0.2887 (3)	0.6327 (2)	0.40342 (14)	0.0677 (14)	
H7	0.3160	0.6952	0.4413	0.081*	
C8	0.6717 (2)	0.5014 (3)	0.62384 (13)	0.0554 (13)	
C9	0.6660 (2)	0.4941 (3)	0.69589 (15)	0.0533 (12)	
H9	0.5959	0.4616	0.7065	0.064*	
C10	0.7652 (2)	0.5355 (3)	0.75212 (11)	0.0478 (11)	
C11	0.8701 (2)	0.5841 (3)	0.73631 (12)	0.0537 (12)	
H11	0.9365	0.6117	0.7739	0.064*	
C12	0.8758 (2)	0.5913 (3)	0.66426 (14)	0.0598 (12)	
H12	0.9460	0.6238	0.6537	0.072*	
C13	0.7766 (3)	0.5500 (3)	0.60803 (11)	0.0590 (13)	
H13	0.7804	0.5548	0.5598	0.071*	
C14	0.7592 (4)	0.5133 (5)	0.8317 (2)	0.0515 (12)	
C15	0.8710 (3)	0.5714 (5)	0.8876 (2)	0.0607 (11)	
H15A	0.8906	0.6596	0.8699	0.073*	
H15B	0.8489	0.5881	0.9327	0.073*	
C16	0.9848 (4)	0.4843 (5)	0.9051 (2)	0.0815 (14)	
H16A	1.0556	0.5437	0.9191	0.098*	
H16B	0.9888	0.4345	0.8616	0.098*	
C17	0.9890 (5)	0.3816 (7)	0.9667 (3)	0.106 (2)	
H17A	1.0702	0.3424	0.9819	0.128*	
H17B	0.9753	0.4311	1.0081	0.128*	
C18	0.8995 (6)	0.2677 (7)	0.9479 (3)	0.110 (2)	
H18A	0.9232	0.2080	0.9133	0.132*	
H18B	0.9047	0.2144	0.9918	0.132*	
C19	0.7500 (4)	0.3574 (4)	0.8414 (2)	0.0680 (12)	
H19A	0.6693	0.3279	0.8140	0.082*	
H19B	0.8084	0.3130	0.8199	0.082*	
C20	0.6444 (4)	0.5822 (5)	0.8442 (2)	0.0762 (13)	
H20A	0.5734	0.5419	0.8105	0.091*	
H20B	0.6403	0.5600	0.8933	0.091*	
C21	0.6361 (5)	0.7371 (6)	0.8352 (3)	0.102 (2)	
H21A	0.7019	0.7795	0.8711	0.153*	
H21B	0.5597	0.7686	0.8416	0.153*	
H21C	0.6415	0.7613	0.7872	0.153*	
C22	0.6905 (6)	0.1909 (6)	0.9190 (3)	0.134 (3)	
H22A	0.6071	0.2201	0.9027	0.200*	
H22B	0.7070	0.1568	0.9681	0.200*	
H22C	0.7048	0.1193	0.8875	0.200*	
C23	0.5058 (4)	0.4166 (5)	0.0171 (2)	0.0467 (12)	
C24	0.34645 (13)	0.44849 (16)	−0.09893 (8)	0.0438 (11)	
C25	0.30502 (17)	0.54244 (19)	−0.15519 (9)	0.0586 (13)	
H25	0.3389	0.6299	−0.1517	0.070*	
C26	0.21302 (16)	0.5056 (2)	−0.21660 (9)	0.0610 (13)	
H26	0.1853	0.5685	−0.2542	0.073*	
C27	0.16244 (15)	0.3748 (2)	−0.22176 (9)	0.0600 (13)	
C28	0.20387 (17)	0.2809 (2)	−0.16550 (10)	0.0635 (13)	
H28	0.1700	0.1934	−0.1689	0.076*	
C29	0.29587 (14)	0.31772 (18)	−0.10409 (9)	0.0564 (13)	
H29	0.3236	0.2549	−0.0664	0.068*	
C30	0.66943 (14)	0.45063 (17)	0.12176 (8)	0.0514 (12)	
C31	0.65863 (16)	0.4641 (2)	0.19300 (8)	0.0484 (11)	
H31	0.5869	0.4986	0.2007	0.058*	
C32	0.75469 (17)	0.4260 (3)	0.25286 (8)	0.0482 (11)	
C33	0.85861 (16)	0.3738 (2)	0.23791 (9)	0.0550 (12)	
H33	0.9244	0.3477	0.2763	0.066*	
C34	0.86610 (14)	0.3595 (3)	0.16610 (10)	0.0638 (14)	
H34	0.9366	0.3235	0.1574	0.077*	
C35	0.77123 (13)	0.3978 (2)	0.10837 (9)	0.0619 (14)	
H35	0.7767	0.3877	0.0607	0.074*	
C36	0.7499 (4)	0.4493 (5)	0.3320 (2)	0.0539 (12)	
C37	0.6310 (4)	0.5130 (5)	0.3376 (2)	0.0749 (14)	
H37A	0.5644	0.4675	0.3027	0.090*	
H37B	0.6218	0.4940	0.3859	0.090*	
C38	0.6175 (5)	0.6667 (6)	0.3245 (3)	0.1006 (18)	
H38A	0.5319	0.6890	0.3051	0.121*	
H38B	0.6595	0.6931	0.2884	0.121*	
C39	0.6695 (7)	0.7485 (7)	0.3945 (4)	0.131 (3)	
H39A	0.6449	0.8436	0.3850	0.157*	
H39B	0.6320	0.7148	0.4313	0.157*	
C40	0.8052 (7)	0.7452 (8)	0.4260 (3)	0.130 (3)	
H40A	0.8434	0.7944	0.3937	0.156*	
H40B	0.8258	0.7924	0.4727	0.156*	
C41	0.8602 (4)	0.5406 (5)	0.3668 (2)	0.0755 (14)	
H41A	0.9337	0.4855	0.3757	0.091*	
H41B	0.8660	0.6123	0.3325	0.091*	
C42	0.7673 (4)	0.3108 (5)	0.3736 (2)	0.0724 (13)	
H42A	0.8410	0.2676	0.3679	0.087*	
H42B	0.7796	0.3296	0.4252	0.087*	
C43	0.6628 (5)	0.2097 (6)	0.3495 (3)	0.0982 (18)	
H43A	0.5914	0.2466	0.3602	0.147*	
H43B	0.6845	0.1245	0.3751	0.147*	
H43C	0.6465	0.1941	0.2979	0.147*	
C44	0.9806 (6)	0.6096 (9)	0.4861 (3)	0.158 (4)	
H44A	1.0120	0.5178	0.4946	0.236*	
H44B	0.9791	0.6510	0.5317	0.236*	
H44C	1.0320	0.6627	0.4637	0.236*	

### Atomic displacement parameters (Å^2^)

**Table:**

	*U*^11^	*U*^22^	*U*^33^	*U*^12^	*U*^13^	*U*^23^
F1	0.0796 (19)	0.085 (2)	0.082 (2)	−0.0066 (17)	−0.0250 (16)	0.0215 (17)
F2	0.0722 (18)	0.099 (3)	0.0657 (17)	0.0042 (17)	−0.0175 (14)	−0.0239 (17)
N1	0.067 (3)	0.035 (2)	0.047 (2)	−0.005 (2)	0.0016 (19)	0.0007 (19)
N2	0.120 (4)	0.083 (3)	0.060 (3)	−0.004 (3)	0.045 (2)	0.011 (2)
N3	0.059 (2)	0.041 (2)	0.043 (2)	−0.0008 (19)	0.0033 (18)	−0.0024 (19)
N4	0.138 (4)	0.102 (4)	0.057 (3)	−0.050 (3)	0.046 (3)	−0.032 (3)
O1	0.067 (2)	0.044 (2)	0.0513 (19)	−0.0015 (17)	−0.0030 (15)	−0.0011 (17)
O2	0.080 (2)	0.050 (2)	0.056 (2)	−0.0115 (19)	−0.0161 (17)	0.0050 (18)
O3	0.077 (2)	0.035 (2)	0.066 (2)	−0.0006 (18)	0.0044 (17)	0.0030 (17)
O4	0.080 (2)	0.038 (2)	0.0494 (19)	0.0007 (18)	−0.0150 (16)	0.0000 (17)
C1	0.055 (3)	0.036 (3)	0.043 (3)	0.002 (3)	0.011 (2)	0.007 (2)
C2	0.050 (3)	0.043 (3)	0.049 (3)	−0.005 (2)	0.010 (2)	0.003 (2)
C3	0.065 (3)	0.056 (3)	0.059 (3)	0.010 (3)	−0.005 (2)	−0.007 (3)
C4	0.076 (4)	0.076 (4)	0.055 (3)	−0.001 (3)	−0.005 (3)	−0.018 (3)
C5	0.053 (3)	0.068 (4)	0.049 (3)	−0.012 (3)	−0.010 (2)	0.019 (3)
C6	0.068 (3)	0.058 (4)	0.072 (3)	−0.001 (3)	−0.014 (3)	0.007 (3)
C7	0.073 (3)	0.052 (4)	0.069 (3)	0.000 (3)	0.003 (3)	−0.006 (3)
C8	0.060 (3)	0.043 (3)	0.055 (3)	−0.005 (2)	0.001 (3)	0.010 (3)
C9	0.050 (3)	0.057 (3)	0.053 (3)	−0.007 (2)	0.013 (2)	0.007 (2)
C10	0.056 (3)	0.043 (3)	0.047 (3)	0.005 (2)	0.016 (2)	0.002 (2)
C11	0.056 (3)	0.060 (3)	0.045 (3)	−0.008 (2)	0.015 (2)	0.007 (2)
C12	0.055 (3)	0.073 (3)	0.050 (3)	−0.007 (3)	0.013 (2)	0.008 (3)
C13	0.078 (3)	0.056 (3)	0.044 (3)	0.003 (3)	0.017 (3)	0.005 (2)
C14	0.050 (3)	0.055 (3)	0.053 (3)	−0.006 (2)	0.019 (2)	−0.005 (2)
C15	0.067 (3)	0.078 (3)	0.040 (2)	−0.006 (3)	0.019 (2)	−0.006 (2)
C16	0.076 (3)	0.117 (4)	0.053 (3)	0.004 (3)	0.019 (2)	0.008 (3)
C17	0.109 (5)	0.149 (6)	0.063 (4)	0.036 (4)	0.026 (3)	0.026 (4)
C18	0.175 (7)	0.102 (5)	0.066 (4)	0.043 (5)	0.054 (4)	0.034 (4)
C19	0.090 (3)	0.067 (3)	0.048 (3)	−0.005 (3)	0.020 (2)	0.003 (2)
C20	0.067 (3)	0.098 (4)	0.071 (3)	0.000 (3)	0.033 (2)	−0.002 (3)
C21	0.120 (4)	0.103 (5)	0.095 (4)	0.043 (4)	0.050 (3)	0.003 (4)
C22	0.229 (7)	0.078 (4)	0.131 (5)	−0.041 (5)	0.113 (5)	−0.002 (4)
C23	0.057 (3)	0.038 (3)	0.041 (3)	0.004 (3)	0.006 (2)	0.002 (2)
C24	0.049 (3)	0.035 (3)	0.045 (2)	0.003 (2)	0.008 (2)	−0.003 (2)
C25	0.065 (3)	0.053 (3)	0.053 (3)	0.005 (3)	0.007 (2)	0.006 (2)
C26	0.060 (3)	0.073 (4)	0.043 (3)	0.008 (3)	0.003 (2)	0.009 (3)
C27	0.048 (3)	0.062 (4)	0.068 (3)	0.006 (3)	0.012 (3)	−0.005 (3)
C28	0.059 (3)	0.045 (3)	0.080 (4)	−0.005 (3)	0.007 (3)	−0.007 (3)
C29	0.063 (3)	0.044 (3)	0.052 (3)	0.003 (3)	−0.002 (2)	0.005 (2)
C30	0.065 (3)	0.038 (3)	0.046 (3)	−0.003 (2)	0.007 (2)	0.004 (2)
C31	0.048 (3)	0.045 (3)	0.050 (3)	−0.003 (2)	0.010 (2)	−0.001 (2)
C32	0.049 (3)	0.047 (3)	0.046 (3)	0.002 (2)	0.009 (2)	0.002 (2)
C33	0.045 (3)	0.067 (3)	0.047 (3)	0.008 (2)	0.001 (2)	0.007 (2)
C34	0.062 (3)	0.078 (4)	0.058 (3)	0.010 (3)	0.028 (2)	−0.013 (3)
C35	0.081 (4)	0.064 (4)	0.039 (3)	0.000 (3)	0.013 (2)	−0.010 (2)
C36	0.068 (3)	0.060 (3)	0.040 (2)	−0.007 (3)	0.024 (2)	−0.003 (2)
C37	0.099 (4)	0.076 (4)	0.062 (3)	0.005 (3)	0.044 (3)	0.005 (3)
C38	0.143 (5)	0.090 (4)	0.094 (4)	0.038 (4)	0.074 (4)	0.013 (3)
C39	0.238 (8)	0.078 (5)	0.111 (6)	0.005 (6)	0.107 (6)	−0.015 (4)
C40	0.232 (9)	0.095 (6)	0.086 (5)	−0.071 (6)	0.084 (6)	−0.046 (4)
C41	0.094 (4)	0.093 (4)	0.045 (3)	−0.018 (3)	0.028 (2)	−0.017 (2)
C42	0.096 (4)	0.074 (3)	0.052 (3)	0.011 (3)	0.028 (2)	0.008 (2)
C43	0.141 (5)	0.070 (4)	0.101 (4)	−0.017 (4)	0.062 (4)	0.010 (3)
C44	0.137 (5)	0.272 (11)	0.069 (4)	−0.103 (6)	0.036 (4)	−0.063 (5)

### Geometric parameters (Å, °)

**Table:**

F1—C5	1.321 (3)	C19—H19B	0.9700
F2—C27	1.329 (3)	C20—C21	1.518 (7)
N1—C1	1.336 (5)	C20—H20A	0.9700
N1—C2	1.409 (4)	C20—H20B	0.9700
N1—H1	0.8600	C21—H21A	0.9600
N2—C19	1.469 (5)	C21—H21B	0.9600
N2—C22	1.472 (6)	C21—H21C	0.9600
N2—C18	1.472 (6)	C22—H22A	0.9600
N3—C23	1.346 (5)	C22—H22B	0.9600
N3—C24	1.411 (4)	C22—H22C	0.9600
N3—H3A	0.8600	C24—C25	1.3900
N4—C40	1.465 (8)	C24—C29	1.3900
N4—C41	1.485 (5)	C25—C26	1.3900
N4—C44	1.491 (7)	C25—H25	0.9300
O1—C1	1.213 (5)	C26—C27	1.3900
O2—C1	1.353 (5)	C26—H26	0.9300
O2—C8	1.382 (3)	C27—C28	1.3900
O3—C23	1.184 (5)	C28—C29	1.3900
O4—C23	1.361 (5)	C28—H28	0.9300
O4—C30	1.390 (3)	C29—H29	0.9300
C2—C3	1.3900	C30—C35	1.3530
C2—C7	1.3900	C30—C31	1.3982
C3—C4	1.3900	C31—C32	1.3999
C3—H3	0.9300	C31—H31	0.9300
C4—C5	1.3900	C32—C33	1.3855
C4—H4	0.9300	C32—C36	1.537 (4)
C5—C6	1.3900	C33—C34	1.3974
C6—C7	1.3900	C33—H33	0.9300
C6—H6	0.9300	C34—C35	1.3688
C7—H7	0.9300	C34—H34	0.9300
C8—C9	1.3900	C35—H35	0.9300
C8—C13	1.3900	C36—C37	1.519 (6)
C9—C10	1.3900	C36—C41	1.536 (6)
C9—H9	0.9300	C36—C42	1.549 (6)
C10—C11	1.3900	C37—C38	1.518 (6)
C10—C14	1.548 (4)	C37—H37A	0.9700
C11—C12	1.3900	C37—H37B	0.9700
C11—H11	0.9300	C38—C39	1.527 (7)
C12—C13	1.3900	C38—H38A	0.9700
C12—H12	0.9300	C38—H38B	0.9700
C13—H13	0.9300	C39—C40	1.500 (8)
C14—C15	1.531 (5)	C39—H39A	0.9700
C14—C19	1.537 (6)	C39—H39B	0.9700
C14—C20	1.543 (5)	C40—H40A	0.9700
C15—C16	1.509 (5)	C40—H40B	0.9700
C15—H15A	0.9700	C41—H41A	0.9700
C15—H15B	0.9700	C41—H41B	0.9700
C16—C17	1.530 (7)	C42—C43	1.517 (6)
C16—H16A	0.9700	C42—H42A	0.9700
C16—H16B	0.9700	C42—H42B	0.9700
C17—C18	1.483 (8)	C43—H43A	0.9600
C17—H17A	0.9700	C43—H43B	0.9600
C17—H17B	0.9700	C43—H43C	0.9600
C18—H18A	0.9700	C44—H44A	0.9600
C18—H18B	0.9700	C44—H44B	0.9600
C19—H19A	0.9700	C44—H44C	0.9600
			
C1—N1—C2	127.6 (4)	H21B—C21—H21C	109.5
C1—N1—H1	116.2	N2—C22—H22A	109.5
C2—N1—H1	116.2	N2—C22—H22B	109.5
C19—N2—C22	109.8 (4)	H22A—C22—H22B	109.5
C19—N2—C18	111.4 (4)	N2—C22—H22C	109.5
C22—N2—C18	110.0 (5)	H22A—C22—H22C	109.5
C23—N3—C24	127.4 (4)	H22B—C22—H22C	109.5
C23—N3—H3A	116.3	O3—C23—N3	129.1 (5)
C24—N3—H3A	116.3	O3—C23—O4	124.5 (4)
C40—N4—C41	112.3 (5)	N3—C23—O4	106.4 (4)
C40—N4—C44	109.2 (5)	C25—C24—C29	120.0
C41—N4—C44	108.0 (5)	C25—C24—N3	116.98 (16)
C1—O2—C8	118.0 (3)	C29—C24—N3	123.00 (16)
C23—O4—C30	117.7 (3)	C26—C25—C24	120.0
O1—C1—N1	127.6 (4)	C26—C25—H25	120.0
O1—C1—O2	123.9 (5)	C24—C25—H25	120.0
N1—C1—O2	108.5 (4)	C25—C26—C27	120.0
C3—C2—C7	120.0	C25—C26—H26	120.0
C3—C2—N1	116.3 (2)	C27—C26—H26	120.0
C7—C2—N1	123.6 (2)	F2—C27—C28	120.20 (19)
C2—C3—C4	120.0	F2—C27—C26	119.78 (19)
C2—C3—H3	120.0	C28—C27—C26	120.0
C4—C3—H3	120.0	C27—C28—C29	120.0
C5—C4—C3	120.0	C27—C28—H28	120.0
C5—C4—H4	120.0	C29—C28—H28	120.0
C3—C4—H4	120.0	C28—C29—C24	120.0
F1—C5—C6	120.5 (3)	C28—C29—H29	120.0
F1—C5—C4	119.5 (3)	C24—C29—H29	120.0
C6—C5—C4	120.0	C35—C30—O4	121.16 (14)
C7—C6—C5	120.0	C35—C30—C31	121.3
C7—C6—H6	120.0	O4—C30—C31	117.23 (14)
C5—C6—H6	120.0	C30—C31—C32	120.7
C6—C7—C2	120.0	C30—C31—H31	119.7
C6—C7—H7	120.0	C32—C31—H31	119.7
C2—C7—H7	120.0	C33—C32—C31	117.0
O2—C8—C9	118.5 (2)	C33—C32—C36	120.75 (19)
O2—C8—C13	121.1 (2)	C31—C32—C36	122.1 (2)
C9—C8—C13	120.0	C32—C33—C34	121.1
C8—C9—C10	120.0	C32—C33—H33	119.5
C8—C9—H9	120.0	C34—C33—H33	119.5
C10—C9—H9	120.0	C35—C34—C33	120.9
C9—C10—C11	120.0	C35—C34—H34	119.5
C9—C10—C14	118.1 (2)	C33—C34—H34	119.5
C11—C10—C14	121.7 (2)	C30—C35—C34	119.0
C12—C11—C10	120.0	C30—C35—H35	120.5
C12—C11—H11	120.0	C34—C35—H35	120.5
C10—C11—H11	120.0	C37—C36—C32	113.3 (3)
C11—C12—C13	120.0	C37—C36—C41	112.0 (4)
C11—C12—H12	120.0	C32—C36—C41	105.5 (3)
C13—C12—H12	120.0	C37—C36—C42	108.3 (3)
C12—C13—C8	120.0	C32—C36—C42	109.8 (3)
C12—C13—H13	120.0	C41—C36—C42	107.8 (4)
C8—C13—H13	120.0	C38—C37—C36	116.4 (4)
C15—C14—C19	110.6 (4)	C38—C37—H37A	108.2
C15—C14—C20	108.3 (3)	C36—C37—H37A	108.2
C19—C14—C20	108.9 (4)	C38—C37—H37B	108.2
C15—C14—C10	112.4 (3)	C36—C37—H37B	108.2
C19—C14—C10	106.1 (3)	H37A—C37—H37B	107.4
C20—C14—C10	110.6 (3)	C37—C38—C39	111.9 (5)
C16—C15—C14	116.8 (4)	C37—C38—H38A	109.2
C16—C15—H15A	108.1	C39—C38—H38A	109.2
C14—C15—H15A	108.1	C37—C38—H38B	109.2
C16—C15—H15B	108.1	C39—C38—H38B	109.2
C14—C15—H15B	108.1	H38A—C38—H38B	107.9
H15A—C15—H15B	107.3	C40—C39—C38	117.0 (5)
C15—C16—C17	112.6 (4)	C40—C39—H39A	108.0
C15—C16—H16A	109.1	C38—C39—H39A	108.0
C17—C16—H16A	109.1	C40—C39—H39B	108.1
C15—C16—H16B	109.1	C38—C39—H39B	108.1
C17—C16—H16B	109.1	H39A—C39—H39B	107.3
H16A—C16—H16B	107.8	N4—C40—C39	113.0 (6)
C18—C17—C16	115.2 (4)	N4—C40—H40A	109.0
C18—C17—H17A	108.5	C39—C40—H40A	109.0
C16—C17—H17A	108.5	N4—C40—H40B	109.0
C18—C17—H17B	108.5	C39—C40—H40B	109.0
C16—C17—H17B	108.5	H40A—C40—H40B	107.8
H17A—C17—H17B	107.5	N4—C41—C36	113.8 (3)
N2—C18—C17	116.1 (6)	N4—C41—H41A	108.8
N2—C18—H18A	108.3	C36—C41—H41A	108.8
C17—C18—H18A	108.3	N4—C41—H41B	108.8
N2—C18—H18B	108.3	C36—C41—H41B	108.8
C17—C18—H18B	108.3	H41A—C41—H41B	107.7
H18A—C18—H18B	107.4	C43—C42—C36	115.3 (4)
N2—C19—C14	116.2 (4)	C43—C42—H42A	108.4
N2—C19—H19A	108.2	C36—C42—H42A	108.4
C14—C19—H19A	108.2	C43—C42—H42B	108.4
N2—C19—H19B	108.2	C36—C42—H42B	108.4
C14—C19—H19B	108.2	H42A—C42—H42B	107.5
H19A—C19—H19B	107.4	C42—C43—H43A	109.5
C21—C20—C14	116.3 (4)	C42—C43—H43B	109.5
C21—C20—H20A	108.2	H43A—C43—H43B	109.5
C14—C20—H20A	108.2	C42—C43—H43C	109.5
C21—C20—H20B	108.2	H43A—C43—H43C	109.5
C14—C20—H20B	108.2	H43B—C43—H43C	109.5
H20A—C20—H20B	107.4	N4—C44—H44A	109.5
C20—C21—H21A	109.5	N4—C44—H44B	109.5
C20—C21—H21B	109.5	H44A—C44—H44B	109.5
H21A—C21—H21B	109.5	N4—C44—H44C	109.5
C20—C21—H21C	109.5	H44A—C44—H44C	109.5
H21A—C21—H21C	109.5	H44B—C44—H44C	109.5
			
C2—N1—C1—O1	1.0 (7)	C24—N3—C23—O3	1.0 (7)
C2—N1—C1—O2	179.4 (3)	C24—N3—C23—O4	−176.9 (3)
C8—O2—C1—O1	−8.6 (6)	C30—O4—C23—O3	12.0 (6)
C8—O2—C1—N1	172.8 (3)	C30—O4—C23—N3	−169.9 (3)
C1—N1—C2—C3	161.6 (3)	C23—N3—C24—C25	−167.6 (3)
C1—N1—C2—C7	−19.3 (5)	C23—N3—C24—C29	14.2 (4)
C7—C2—C3—C4	0.0	C29—C24—C25—C26	0.0
N1—C2—C3—C4	179.1 (3)	N3—C24—C25—C26	−178.30 (16)
C2—C3—C4—C5	0.0	C24—C25—C26—C27	0.0
C3—C4—C5—F1	−179.8 (3)	C25—C26—C27—F2	178.44 (18)
C3—C4—C5—C6	0.0	C25—C26—C27—C28	0.0
F1—C5—C6—C7	179.8 (3)	F2—C27—C28—C29	−178.43 (18)
C4—C5—C6—C7	0.0	C26—C27—C28—C29	0.0
C5—C6—C7—C2	0.0	C27—C28—C29—C24	0.0
C3—C2—C7—C6	0.0	C25—C24—C29—C28	0.0
N1—C2—C7—C6	−179.1 (3)	N3—C24—C29—C28	178.19 (17)
C1—O2—C8—C9	112.9 (3)	C23—O4—C30—C35	72.2 (3)
C1—O2—C8—C13	−74.7 (4)	C23—O4—C30—C31	−113.7 (3)
O2—C8—C9—C10	172.4 (3)	C35—C30—C31—C32	1.6
C13—C8—C9—C10	0.0	O4—C30—C31—C32	−172.52 (15)
C8—C9—C10—C11	0.0	C30—C31—C32—C33	−0.8
C8—C9—C10—C14	−174.6 (3)	C30—C31—C32—C36	175.4 (3)
C9—C10—C11—C12	0.0	C31—C32—C33—C34	−0.2
C14—C10—C11—C12	174.4 (3)	C36—C32—C33—C34	−176.4 (2)
C10—C11—C12—C13	0.0	C32—C33—C34—C35	0.5
C11—C12—C13—C8	0.0	O4—C30—C35—C34	172.55 (16)
O2—C8—C13—C12	−172.2 (3)	C31—C30—C35—C34	−1.3
C9—C8—C13—C12	0.0	C33—C34—C35—C30	0.3
C9—C10—C14—C15	−176.6 (3)	C33—C32—C36—C37	177.4 (3)
C11—C10—C14—C15	8.9 (4)	C31—C32—C36—C37	1.5 (4)
C9—C10—C14—C19	62.5 (4)	C33—C32—C36—C41	54.6 (4)
C11—C10—C14—C19	−112.0 (3)	C31—C32—C36—C41	−121.4 (3)
C9—C10—C14—C20	−55.4 (4)	C33—C32—C36—C42	−61.4 (4)
C11—C10—C14—C20	130.1 (3)	C31—C32—C36—C42	122.7 (3)
C19—C14—C15—C16	38.0 (5)	C32—C36—C37—C38	−78.6 (5)
C20—C14—C15—C16	157.2 (4)	C41—C36—C37—C38	40.5 (5)
C10—C14—C15—C16	−80.3 (5)	C42—C36—C37—C38	159.3 (4)
C14—C15—C16—C17	−87.6 (5)	C36—C37—C38—C39	−87.5 (5)
C15—C16—C17—C18	69.6 (6)	C37—C38—C39—C40	68.5 (8)
C19—N2—C18—C17	72.7 (6)	C41—N4—C40—C39	75.8 (6)
C22—N2—C18—C17	−165.2 (4)	C44—N4—C40—C39	−164.4 (5)
C16—C17—C18—N2	−52.7 (7)	C38—C39—C40—N4	−52.7 (8)
C22—N2—C19—C14	143.9 (4)	C40—N4—C41—C36	−95.9 (6)
C18—N2—C19—C14	−94.0 (5)	C44—N4—C41—C36	143.6 (5)
C15—C14—C19—N2	44.3 (5)	C37—C36—C41—N4	41.0 (5)
C20—C14—C19—N2	−74.6 (5)	C32—C36—C41—N4	164.6 (4)
C10—C14—C19—N2	166.4 (4)	C42—C36—C41—N4	−78.1 (5)
C15—C14—C20—C21	61.0 (5)	C37—C36—C42—C43	55.2 (5)
C19—C14—C20—C21	−178.7 (4)	C32—C36—C42—C43	−68.9 (5)
C10—C14—C20—C21	−62.5 (5)	C41—C36—C42—C43	176.6 (4)

### Hydrogen-bond geometry (Å, °)

**Table:**

*D*—H···*A*	*D*—H	H···*A*	*D*···*A*	*D*—H···*A*
N3—H3A···O3^i^	0.86	2.15	3.007 (5)	177
N1—H1···O1^ii^	0.86	2.16	3.002 (5)	167

Symmetry codes: (i) −*x*+1, *y*+1/2, −*z*; (ii) −*x*+1, *y*−1/2, −*z*+1.
